# A Glucose Metabolism‐Modulatory Nanobiohybrid Vaccine Platform Promotes Anti‐Tumor Immunity by Orchestrating Autophagy‐Dependent Cross‐Presentation and H_2_S‐Enhanced NLRP3 Inflammasome Signaling

**DOI:** 10.1002/advs.76654

**Published:** 2026-07-21

**Authors:** Weidong Wang, Yimin Gong, Jianing Li, Tianze Wu, Mingli Deng, Yannan Yang

**Affiliations:** ^1^ Shanghai Key Laboratory of Molecular Catalysis and Innovative Materials Department of Chemistry Fudan University Shanghai China; ^2^ State Key Laboratory of Genetics and Development of Complex Phenotypes Obstetrics and Gynecology Hospital Children's Hospital Fudan University Shanghai China; ^3^ South Australian Immunogenomics Cancer Institute The University of Adelaide Adelaide South Australia Australia

**Keywords:** autophagy, cancer immunotherapy, hydrogen sulfide, nanobiohybrids, NLRP3 inflammasome

## Abstract

The clinical translation of cancer vaccines remains limited by the inability to effectively coordinate innate and adaptive immunity. Leveraging the metabolism pattern of the immune system, herein, we report a glucose metabolism‐modulatory nanobiohybrid vaccine with superior antitumor immunity via orchestrating cross‐presentation‐enhanced adaptive immunity and proinflammatory signaling‐mediated innate immunity. This nanobiohybrid vaccine is constructed by a nanoscale hydrogen‐bonded organic framework (HOF) co‐encapsulating glucose oxidase (GOx), and a model antigen, ovalbumin (OVA) (denoted OVA/GOx@HOF). OVA/GOx@HOF enables a GOx‐mediated enzyme reaction that consumes glucose and produces H_2_O_2_ within dendritic cells (DCs) in controlled kinetics, which disrupts intracellular glucose metabolism and causes redox imbalance but minimally impacts cell viability. These effects induce autophagic machinery in DCs that promotes antigen cross‐presentation, thereby boosting cytotoxic T cell response. Meanwhile, through RNA sequencing, we found that OVA/GOx@HOF stimulates innate immunity through an NF‐κB signaling‐mediated and endogenous hydrogen sulfide (H_2_S)‐enhanced NLRP3 inflammasome pathway, leading to significant DC maturation. In a melanoma tumor model, this nanobiohybrid vaccine effectively inhibits tumor growth and metastasis, and can be applied to encapsulate neoantigens to generate a potent personalized cancer vaccine. Our findings suggested the remarkable potential of enzyme‐driven nanobiohybrid materials as a metabolism‐modulatory platform for cancer vaccines.

## Graphical abstract

A nanobiohybrid vaccine was designed by constructing a hydrogen‐bonded organic framework coencapsulated with glucose oxidase (GOx) and tumor antigens. This nanobiohybrid vaccine executed a GOx‐catalyzed enzyme reaction that disrupted glucose metabolism and induced redox imbalance in dendritic cells, leading to autophagy‐dependent antigen cross‐presentation and H_2_Senhanced NLRP3 inflammasome signaling, thereby promoting DC maturation and cytotoxic T cell responses that suppressed tumor progression.

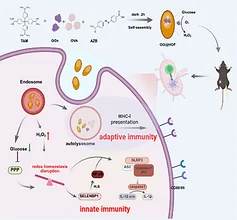

## 1 Introduction

Vaccines have emerged as a new‐generation strategy for cancer treatment owing to a wide range of advantages, including high specificity, durable anticancer activity, and minimal adverse effects [1, 2]. Despite extensive efforts made to improve the therapeutic efficacy of cancer vaccines, such as developing immunostimulatory adjuvants and advanced antigen delivery systems [3, 4], the clinical outcomes remain largely unsatisfactory compared to current first‐line treatments, hindering their widespread adoption. Challenges still exist in coordinating the innate (e.g., proinflammatory signaling) and adaptive immunity (e.g., antigen presentation and T cell activation) of the immune system, which are essential to achieving a maximized antitumor immune response [5, 6].

Antigen‐presenting cells (APCs) play a central role in coordinating both the arms of innate and adaptive immunity, and the initiation of the latter is intrinsically linked to the effective activation of the former [7, 8, 9]. The activation of multiprotein complexes like the NLRP3 inflammasome represents a crucial innate immune pathway. Upon sensing cellular stress or danger signals, NLRP3 activation triggers the release of pro‐inflammatory cytokines such as IL‐1β and IL‐18, which are pivotal for driving T cell responses [10, 11, 12]. Concurrently, the emerging field of immunometabolism has revealed that intracellular metabolic reprogramming in DCs is a fundamental regulator of their function, where shifts in glucose metabolism can directly influence intracellular redox balance/oxidative stress, antigen presentation, and T cell priming efficacy [13, 14, 15, 16]. Moreover, prolonged disruption of glucose supply can lead to nutrient deprivation that in turn triggers autophagy, a cellular protective measure in response to a nutrient‐deficient environment. It has been reported that autophagy in APCs can induce an autophagosome‐mediated antigen cross‐presentation on MHC‐I receptors to promote adaptive immune activation [17, 18]. Building on the sophisticated role of glucose metabolism and the interplay of the relevant immune signaling pathways, we hypothesize that targeting glucose metabolic and inflammatory pathways in APCs would be a new immunoactivation strategy for coordinating the innate and adaptive immune signaling and inducing favorable antitumor T cell immunity.

The design of nanobiohybrids integrates synthetic materials and the function of biosystems. Recent progress has shown that nanobiohybrids are promising candidates for protein delivery, although their application in the vaccine field has been largely explored [19, 20, 21]. In this work, we introduce a glucose metabolism‐modulatory nanobiohybrid platform for cancer vaccine. This nanobiohybrid vaccine was constructed by synthesizing nanoscale HOF encapsulated with GOx and OVA (a model antigen) (OVA/GOx@HOF), which targets glucose metabolism and orchestrates multiple innate and adaptive immune activation mechanisms (Figure 1a,b). Firstly, the catalytic activity of GOx consumed intracellular glucose, creating a nutrient‐restricted state of DCs that triggered autophagy, a process linked to MHC I antigen cross‐presentation and CD8+ T cell responses. Secondly, OVA/GOx@HOF induced intracellular redox imbalance and endogenous H_2_S production, which synergistically triggered NF‐κB‐mediated proinflammatory NLRP3 inflammasome signaling, leading to considerably enhanced DC maturation that promotes T cell activation. We demonstrated that the nanobiohybrid vaccine could activate robust T cell immunity and effectively inhibit tumor growth and metastasis in a melanoma tumor model, and that this platform could be extended to a personalized cancer vaccine for delivering neoantigens, showing impressive anti‐tumour performance in synergy with immune checkpoint blockades.

**FIGURE 1:**
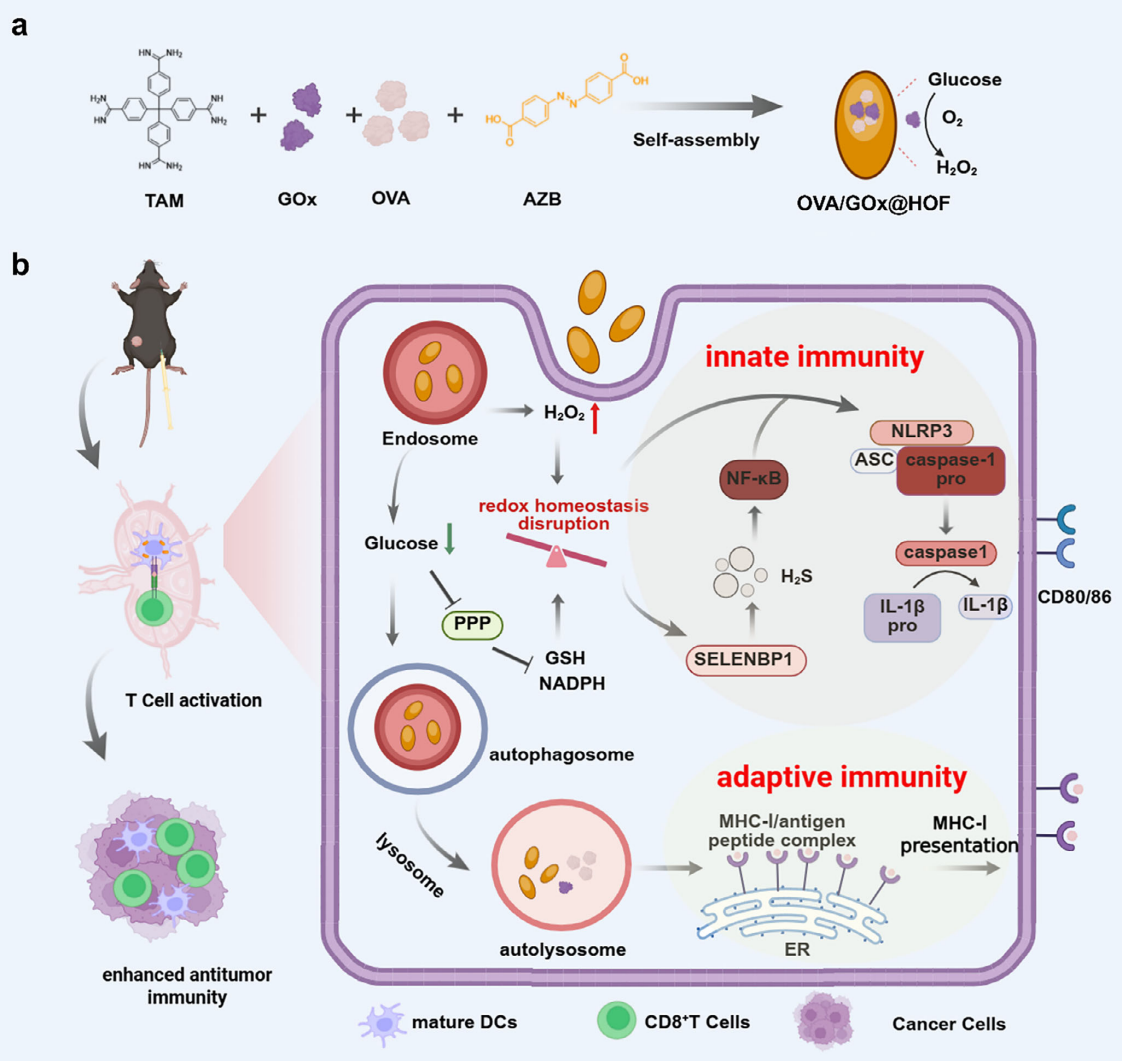
Schematic illustration of the fabrication and mechanism of action of OVA/GOx@HOF. (a) Fabrication procedure of OVA/GOx@HOF. (b) OVA/GOx@HOF activates the H_2_S‐enhanced NF‐κB/NLRP3 inflammasome pathway (innate immunity) and induces autophagy‐mediated antigen cross‐presentation (adaptive immunity) to induce an antitumor immune response.

## 2 Results and Discussions

### 2.1 Fabrication of a Nanobiohybrid Platform for Antigen Delivery

The nanoscale HOF was formed by the molecular assembly of azobenzenedicarboxylate (AZB) and tetrakis(4‐amidiniumphenyl) methane (TAM) hydrogen bonding interactions, as described in the literature (Figure 2a) [22, 23]. OVA and GOx were co‐encapsulated in situ to form the nanobiohybrid vaccine OVA/GOx@HOF. Transmission electron microscopy (TEM) images revealed that OVA/GOx@HOF retained a rod‐like morphology similar to that of the pristine HOF, with an average length of ∼200 nm and a width of ∼30 nm (Figure 2b). Fourier‐Transform Infrared (FTIR) analysis verified the successful preparation of the OVA/GOx@HOF nanobiohybrid (Figure 2c). The characteristic C═O (1695 cm^−1^) and C═N (1685 cm^−1^) stretching vibrations exhibited reduced intensity, consistent with the establishment of a hydrogen‐bonded rigid network that limits molecular vibration. Detected by HPLC, OVA/GOx@HOF the encapsulation efficiency of GOx and OVA in OVA/GOX@HOF were 5% and 13%, respectively (Figure S1).

**FIGURE 2:**
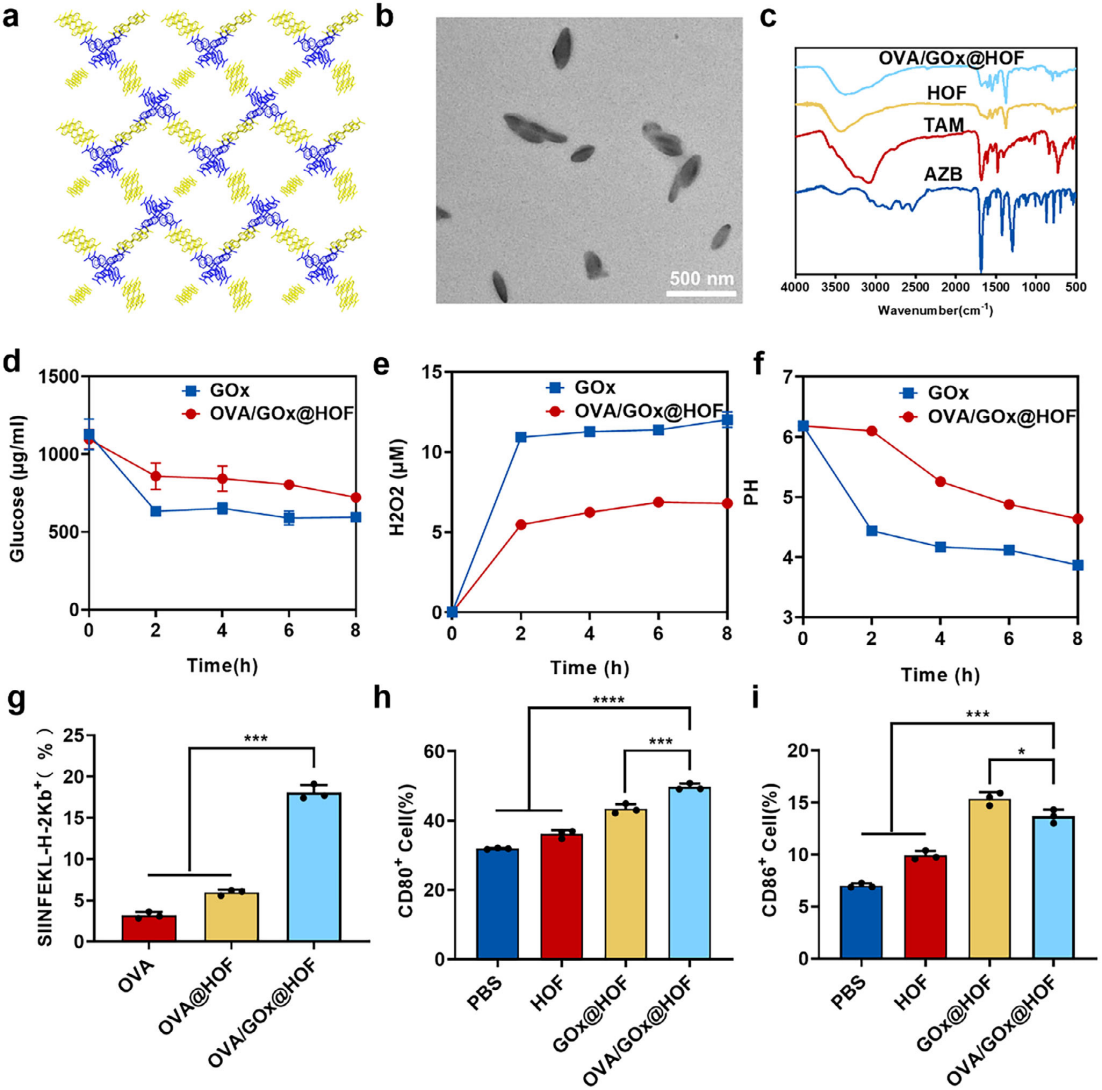
Preparation, characterization, and DC activation of nanovaccines. (a) 3D structural diagram of OVA/GOx@HOF. (b) TEM images of the OVA/GOx@HOF. (c) FTIR spectrum of TAM, AZB, HOF, and OVA/GOx@HOF. (d–f) Kinetic profiles of free GOx and OVA/GOx@HOF in glucose solution (1 mg mL^−^^1^), monitoring (d) glucose depletion, (e) H_2_O_2_ generation, and (f) pH (n = 3). (g–i) Statistical data showing OVA antigen cross‐presentation (g) and CD80+ (h), CD86+ (i) efficiencies in DC2.4 treated with NPs for 24 h (n =  3). The OVA content was fixed at 10 µg mL^−1^. Data are presented in the form of means ± SEM. *P* values were determined by Student's t‐test and one‐way ANOVA, ^***^ *p* < 0.001.

It is well documented that the azobenzene moiety can be cleaved by diverse cellular reductases within a hypoxic milieu, where the expression of such enzymes is frequently upregulated [24, 25]. In this system, GOx enzymatic activity triggers hypoxia by oxygen consumption, thereby enabling the intracellular activation of reductases. These enzymes, in turn, cleave the azo bond within OVA/GOx@HOF, triggering the self‐disassembly of the nanohybrid structure. To monitor protein release, we encapsulated fluorescein isothiocyanate‐labeled ovalbumin (OVA‐FITC) as a model cargo within the HOF. When a chemically reducing environment, simulating intracellular hypoxia, was introduced using sodium dithionite (Na_2_S_2_O_4_, 20 mM) [26], the HOF matrix began to degrade within 12 h, as visualized by TEM (Figure S2). After 48 h, the framework was nearly completely disrupted, yielding abundant structural fragments. Correspondingly, over 80% of the encapsulated OVA‐FITC was released from the nanobiohybrid within the same period (Figure S3), confirming efficient payload release in response to reductive conditions.

To evaluate the catalytic activity of glucose oxidase (GOx) after encapsulation, the enzymatic kinetics of OVA/GOx@HOF were investigated by monitoring glucose consumption and H_2_O_2_ generation. As shown in Figure 2d–f, OVA/GOx@HOF exhibited markedly attenuated enzymatic kinetics, with glucose consumption and H_2_O_2_ production reduced to 30.0% and 43.4% of those of free GOx, respectively. The decelerated reaction can be ascribed to steric confinement within the HOF matrix that restricts substrate diffusion. This reduced enzyme reaction kinetics is important to alleviate cytotoxic effects associated with excessive H_2_O_2_ generation. The cytotoxicity of OVA/GOx@HOF was evaluated using an MTT assay, revealing negligible toxicity at concentrations up to 25 µg mL^−1^. Based on these results, further investigations were conducted using the material at 12.5 µg mL^−1^ (Figure S4).

The maturation state of dendritic cells (DCs) is a prerequisite for effective antigen presentation and the subsequent orchestration of adaptive immunity [27, 28]. We next studied the interactions between OVA/GOx@HOF and DCs. Interestingly, compared to other groups, OVA/GOx@HOF significantly promoted DC maturation, as evidenced by the upregulated expression of costimulatory factors (CD80 and CD86) in DC2.4 cells (Figure 2h,i). Moreover, OVA/GOx@HOF treatment led to the highest level of SIINFEKL peptide–MHC I complex presentation on DC surfaces (Figure 2g and Figure S5), confirming its strong ability to induce antigen cross‐presentation. Analysis of mean fluorescence intensity (MFI) confirmed a significant upregulation in the surface density of SIINFEKL‐MHC I complex, CD80, and CD86 on DC2.4 cells treated with OVA/GOx@HOF, further substantiating its potent ability to induce both cross‐presentation and maturation (Figure S15).

### 2.2 OVA/GOx@HOF Modulates Glucose Metabolism and Intracellular Redox Homeostasis and Promotes Autophagy‐Dependent Antigen Cross‐Presentation

The pentose phosphate pathway (PPP) is a key route of glucose catabolism that generates nicotinamide adenine dinucleotide phosphate (NADPH) to support detoxification and maintain intracellular redox homeostasis, thereby playing a critical role in regulating cell growth [29, 30]. The controlled perturbation of intracellular redox homeostasis is synergistically enhanced by two mechanisms: the generation of hydrogen peroxide (H_2_O_2_) by OVA/GOx@HOF, coupled with reduced flux through the pentose phosphate pathway (PPP) due to glucose depletion. This combined action elevates the level of intracellular oxidative stress. To evaluate OVA/GOx@HOF, we assessed its impact on the PPP throughput. OVA/GOx@HOF‐treated cells were processed, and the levels of PPP‐related molecules, including glucose‐6‐phosphate (G6P), NADPH, and glutathione (GSH), were detected. As shown in Figure 3a–c, after co‐incubation with OVA/GOx@HOF for 12 h, G6P decreased to 10 µmL^−1^. As expected, a significant reduction in the NADPH/NADP^+^ ratio was recorded at 12 h, suggesting pentose phosphate pathway (PPP) inhibition and a consequent disruption of the inherent redox balance. The observed plunge in glutathione (GSH) levels to a mere 7% of the baseline value and the significant reduction in the GSH/GSSG ratio (Figure S17) provided further evidence for a severe disruption in redox homeostasis. The strong green fluorescence signal of 2', 7'‐dichlorodihydrofluorescein diacetate (DCFH‐DA) also confirmed the increase of intracellular oxidative stress (Figure 3d,e).

**FIGURE 3:**
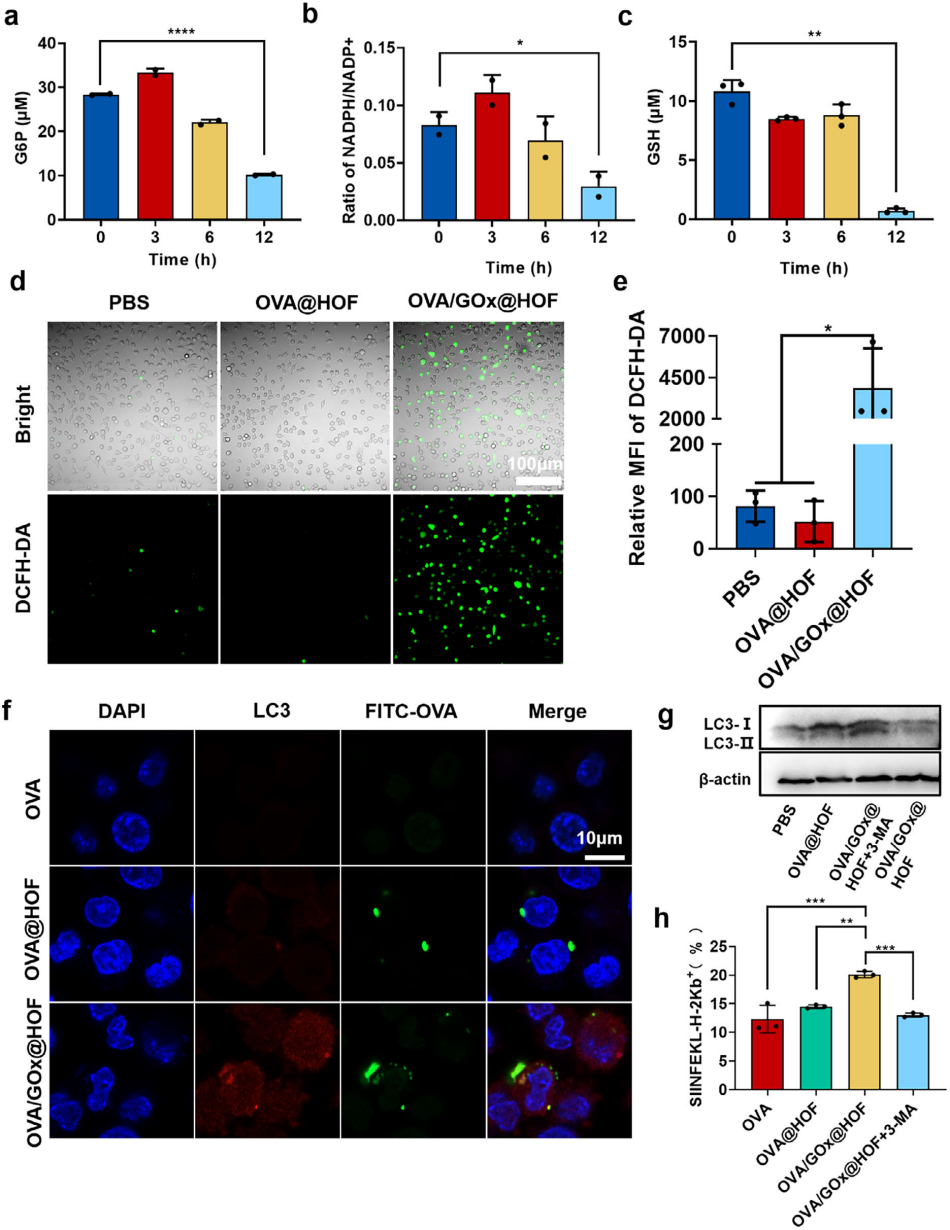
OVA/GOx@HOF modulated glucose metabolism and intracellular redox homeostasis and promoted autophagy‐dependent antigen cross‐presentation. (a) Intracellular G6P concentration at indicated time points after OVA/GOx@HOF treatment (n = 2). (b) Cellular NADPH/NADP^+^ ratio at indicated time points after OVA/GOx@HOF treatment (n = 2). (c) Intracellular GSH levels at indicated time points after OVA/GOx@HOF treatment (n = 3). (d) Confocal microscopy images showing intracellular ROS levels in DC2.4 cells following 24 h treatment with OVA/GOx@HOF under low‐glucose conditions. (e) Mean fluorescence intensity (MFI) of the ROS probe DCFH‐DA in treated DC2.4 cells (n = 3). (f) Representative confocal image showing DCs internalizing OVA/GOx@HOF nanoparticles (green) and stained for the autophagy marker LC3 (red); nuclei are labeled with Hoechst 33342 (blue). (g) Representative Western blot images reveal LC3 expression in DC2.4 cells following incubation with different formulations. (h) Flow cytometric analysis of OVA antigen cross‐presentation level in DC2.4 cells after incubation with different formulations after 24 h treatment (n =  3). Data are presented in the form of means ± SD. *P* values were determined by Student's t‐test and one‐way ANOVA, ^*^ *p* < 0.05, ^**^ *p* < 0.01, and ^***^ *p* < 0.001.

It is known that nutrient starvation and oxidative damage can both lead to autophagy [31]. Recent reports have shown that autophagy of antigen‐presenting cells promotes cross‐presentation of antigens [32, 33]. Based on the aforementioned observations on the glucose consumption and oxidative stress, we explored whether OVA/GOx@HOF could induce autophagy in DCs. Confocal microscopy results showed that, after 12 h incubation, most OVA colocalized with the autophagosome marker LC3 (Figure 3f), but not with lysosomes (Figure S6). This result indicated that OVA/GOX@HOF can induce autophagosome formation and effectively deliver antigens into these compartments. Western blot results showed the conversion of LC3‐I to LC3‐II upon OVA/GOx@HOF treatment, and such conversion was inhibited by treating DCs with an autophagy inhibitor, 3‐methyladenine (3‐MA) (Figure 3g). These results confirmed the successful induction of autophagy by OVA/GOx@HOF. Moreover, we found that OVA/GOx@HOF induced significant MHC‐I cross‐presentation of OVA antigens OVA/GOx@HOF in DC cells, while the addition of 3‐MA significantly inhibited such effects (Figure 3h), confirming an autophagy‐dependent antigen cross‐presentation mechanism.

Similarly, the above results were validated in BMDC cells, as shown in Figure S16a‐d, we observed a significant decrease in the G6P, GSH/GSSG, NADPH/NADP^+^ ratio, and an increase in ROS levels (DCFH‐DA probe), consistent with our findings in DC2.4 cells. BMDCs treated with OVA/GOx@HOF exhibited significantly higher expression of CD80, CD86, and SIINFEKL‐H‐2Kb compared to controls (Figure S16e,f), confirming that the nanovaccine promotes maturation and cross‐presentation in primary cells. Western blot analysis of BMDCs confirmed the enhanced conversion of LC3‐I to LC3‐II upon OVA/GOx@HOF treatment (Figure S16g). This further demonstrates the immune activation pathway mediated by our OVA/GOx@HOF‐based nanovaccine.

To further establish the functional role of autophagy in DC maturation and subsequent T cell priming, we treated DCs with the autophagy inhibitor 3‐methyladenine prior to OVA/GOx@HOF stimulation. As shown in Figure S22, the OVA/GOx@HOFinduced upregulation of the costimulatory molecules CD80 and CD40, as well as MHCII, was significantly attenuated in the presence of 3MA, indicating that autophagy is essential for full DC maturation. Moreover, it has been well established that mature DCs are critical for priming antigen‐specific T cell responses [34, 35]. The observed reduction in costimulatory and MHC molecules upon 3MA treatment therefore strongly suggests that autophagy is a critical upstream event for T cell priming in our system.

### 2.3 OVA/GOx@HOF Enhanced NLRP3 Activation and Endogenous Hydrogen Sulfide Production

We next focused on innate immunity and sought to gain in‐depth insights into the mechanism of OVA/GOx@HOF‐mediated DCs maturation. As indicated by the RNA sequencing results, Gene Ontology (GO) functional enrichment analysis of the upregulated differentially expressed genes in the PBS group and the OVA/GOx@HOF group identified a total of 355 significantly enriched terms. Among these, 266 were associated with Biological Process (BP), while 34 and 55 were related to Cellular Component (CC) and Molecular Function (MF), respectively. Immune‐related BP terms primarily included cellular response to interleukin‐1, cellular response to interferon‐gamma, regulation of tumor necrosis factor production, regulation of inflammatory response, regulation of production of molecular mediator of immune response, regulation of toll‐like receptor signaling pathway, and regulation of innate immune response (Figure 4a). The cellular response to OVA/GOx@HOF, as determined by KEGG enrichment analysis, robustly activated several innate immune and metabolic pathways, comprising sulfur metabolism, NOD‐like receptor signaling, cytokine‐cytokine receptor interaction, and NF‐κB signaling. These pathways are known to mediate a series of downstream immune and inflammatory cascades, as well as metabolic regulation (Figure 4b). It has been reported that the NF‐κB pathway participates in regulating the composition of the downstream NLRP3 inflammasome [36, 37].

**FIGURE 4:**
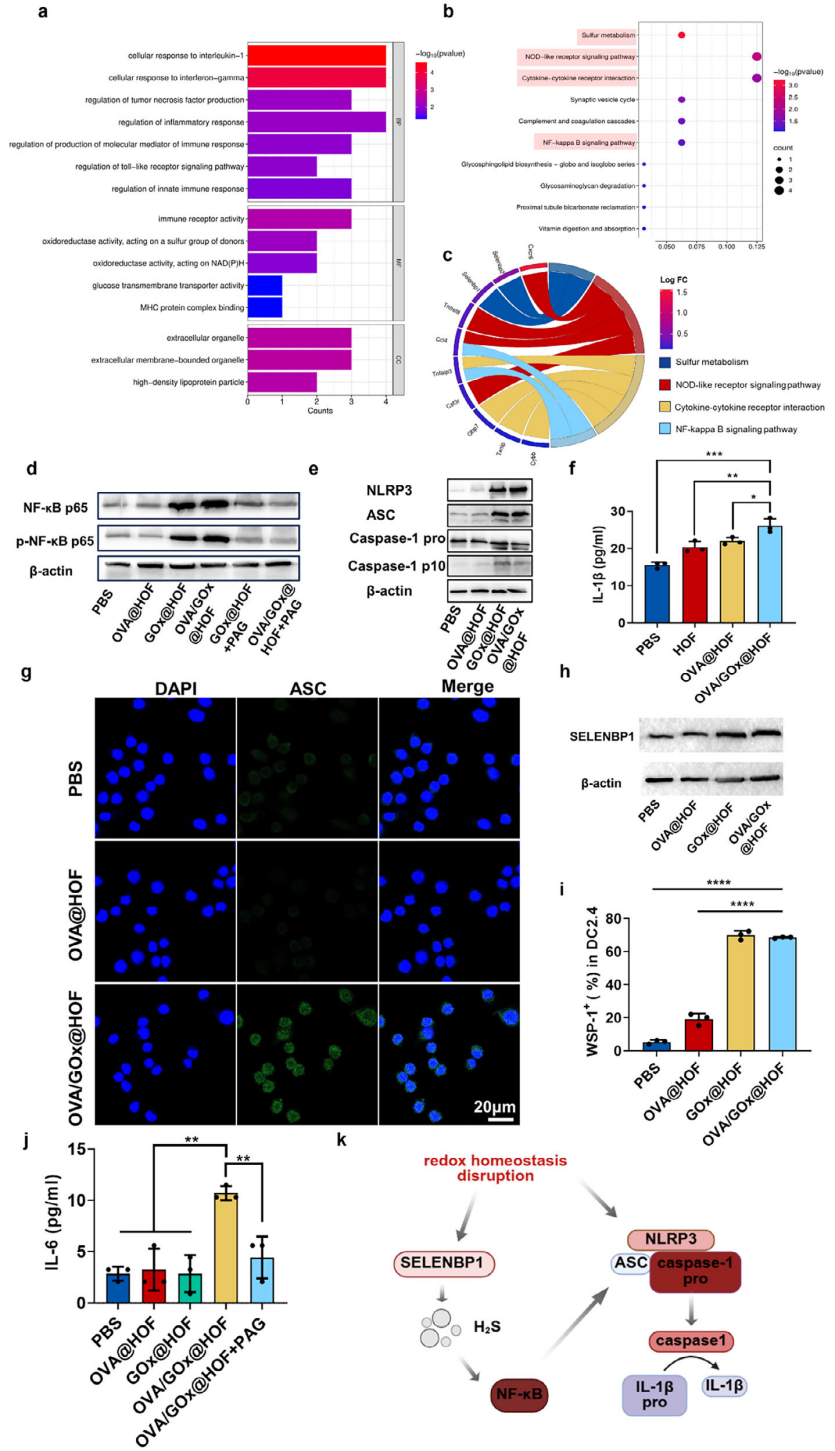
OVA/GOx@HOF induced endogenous H_2_S‐enhanced NLRP3 inflammasome signaling. a‐c) Transcriptomic analysis of DC2.4 after treatment with PBS or OVA/GOx@HOF: (a) Gene Ontology (GO) terms enriched for upregulated differentially expressed genes; (b) Kyoto Encyclopedia of Genes and Genomes (KEGG) pathways enriched for upregulated differentially expressed genes; (c) Chord diagram of sulfur metabolism, NOD receptor pathway, cytokine receptor, and NF kappa B pathway. (d) Representative Western blot images reveal NF‐κB expression in DC2.4 cells following incubation with different formulations. (e) Representative Western blot images reveal NLRP3, ASC, and Caspase‐1 expression in DC2.4 cells following incubation with different formulations. (f) IL‐1β concentrations in supernatants of DC2.4 cells treated with various formulations after 24 h treatment (n = 3). (g) Confocal images display ASC spots in DC2.4 cells. The cell nucleus was stained with DAPI and displayed in blue; ASC is displayed in green. (h) Representative Western blot images reveal SELENBP1 expression in DC2.4 cells following incubation with different formulations. (i) Flow cytometric analysis of H2S levels in DC2.4 cells after incubation with different formulations after 24 h treatment. (j) IL‐6 concentrations in supernatants of DC2.4 cells after incubating with different formulations after 24 h treatment (n = 3). (k) Scheme diagram for the pathway of NLRP3. Data in f, i, and j are presented in the form of means ± SD. *P* values were determined by one‐way ANOVA, ^*^ *p* < 0.05, ^**^ *p* < 0.01, ^***^ *p* < 0.001, and ^****^ *p* < 0.0001.

Based on the RNA sequencing analysis, we conducted further investigations to confirm the pro‐inflammatory pathways involved. Western blot results showed increased protein expression of NF‐κB in the OVA/GOx@HOF group (Figure 4d). Treatment with OVA/GOx@HOF led to increased protein expression of NLRP3 and ASC (Figure 4e), alongside enhanced release of the cytokines IL‐1β and IL‐6 (Figure 4f and Figure S8). Confocal microscopy analysis revealed an increase in ASC speck formation in the OVA/GOx@HOF treated group (Figure 4g), indicating that OVA/GOx@HOF activates the NLRP3 inflammasome via the NF‐κB pathway. Importantly, this inflammasome activation correlated with the enhanced DC maturation and antigen cross‐presentation observed in Figure 2g–i, where OVA/GOx@HOF treatment significantly upregulated the expression of costimulatory molecules CD80 and CD86 as well as the SIINFEKL–MHC I complex on DCs. Furthermore, addition of the NLRP3 inhibitor, MCC950, inhibited the maturation of DC2.4 cells, further substantiating that OVA/GOx@HOF induces DC maturation through the NLRP3 inflammasome pathway (Figure S7).

As shown in Figure 4c, the genes enriched in the aforementioned pathways mainly included SELENBP1 and CCL4, among others, which may play principal roles within the relevant signaling pathways. Selenium‐binding protein 1 (SELENBP1) functions in redox regulation, cellular metabolism, and disease pathogenesis, and is a member of a distinct family of selenium‐binding proteins [38, 39]. Furthermore, SELENBP1 functions as a methanethiol oxidase (MTO), participating in hydrogen sulfide (H_2_S) generation. Previous studies have indicated that H_2_S promotes the assembly of the NLRP3 inflammasome and the progression of inflammation via NF‐κB [40, 41, 42]. To investigate whether H_2_S enhances the downstream pro‐inflammatory response, we assessed its immunostimulatory mechanisms using western blot, flow cytometry, and enzyme‐linked immunosorbent assay (ELISA). As shown in Figure 4h, the results revealed that OVA/GOx@HOF enhanced SELENBP1 protein expression in DCs. WSP‐1 is a selective and rapid‐response fluorescent probe specific for H_2_S. WSP‐1 reacts with H_2_S and releases a fluorophore. As depicted in Figure 4i, compared to the PBS group, treatment with OVA/GOx@HOF resulted in a 3.6‐fold increase in the WSP‐1 fluorescence signal in DCs, confirming that OVA/GOx@HOF treatment promotes the production of endogenous H_2_S (Figure S9). The addition of the H_2_S inhibitor, PAG, reduced the protein expression of NF‐κB in DCs and decreased the release of pro‐inflammatory factors (Figure 4d,j). The above findings indicated that OVA/GOx@HOF regulates the endogenous H_2_S‐enhanced NLRP3‐mediated inflammatory response (Figure 4k).

To establish causality between increased H_2_S production and NLRP3 inflammasome activation, we treated DC2.4 cells with the specific H_2_S synthesis inhibitor DLpropargylglycine (PAG) prior to OVA/GOx@HOF exposure. As shown in Figure S23, PAG cotreatment significantly reduced OVA/GOx@HOFinduced upregulation of NLRP3, ASC, and cleaved Caspase1 (p10), demonstrating that endogenous H_2_S is essential for full inflammasome activation. Flow cytometric analysis revealed that PAG treatment significantly attenuated the OVA/GOx@HOF induced upregulation of CD80, CD40, and MHCII, as well as the surface expression of the SIINFEKL–H‐2Kb complex. This result indicates that inhibition of hydrogen sulfide production reduces DC maturation and antigen cross‐presentation. Collectively, these results establish a causal relationship wherein OVA/GOx@HOFinduced endogenous H_2_S enhances NLRP3 inflammasome activation, which in turn promotes DC maturation, cross‐presentation, and immune response priming.

### 2.4 OVA/GOx@HOF Enhanced Antitumor and Anti‐Metastasis Immunity in Melanoma Tumor Model

We established a B16‐OVA melanoma‐bearing mouse model to evaluate the immunotherapy efficacy of OVA/GOx@HOF. Firstly, we tested OVA/GOx@HOF its ability to target lymph nodes. OVA/GOx@HOF was conjugated with Cy5 to track their in vivo distribution. As shown in Figure 5a,b, after 24 h, subcutaneous injection of OVA/GOx@HOF and OVA/GOx@HOF‐Cy5 showed high fluorescence intensity in the popliteal lymph node on the administered side, indicating effective accumulation of OVA/GOx@HOF. To identify the primary cellular targets of OVA/GOx@HOF following subcutaneous administration, we performed an in vivo uptake study using OVA/GOx@HOF‐Cy5. B16‐OVA tumor‐bearing mice were subcutaneously injected with OVA/GOx@HOF‐Cy5, and draining lymph nodes (dLNs) and tumors were collected 24 h postinjection for flow cytometric analysis. As shown in Figure S18 and Figures S34–S36, the Cy5 signal was predominantly detected within CD11c^High^ dendritic cells in dLNs. These results demonstrate that OVA/GOx@HOF is selectively taken up by DCs in vivo, with minimal direct engagement of macrophages or tumor cells. This selective targeting is consistent with the high abundance of DCs in the subcutaneous compartment and their specialized antigen‐capturing function [27, 43, 44].

**FIGURE 5:**
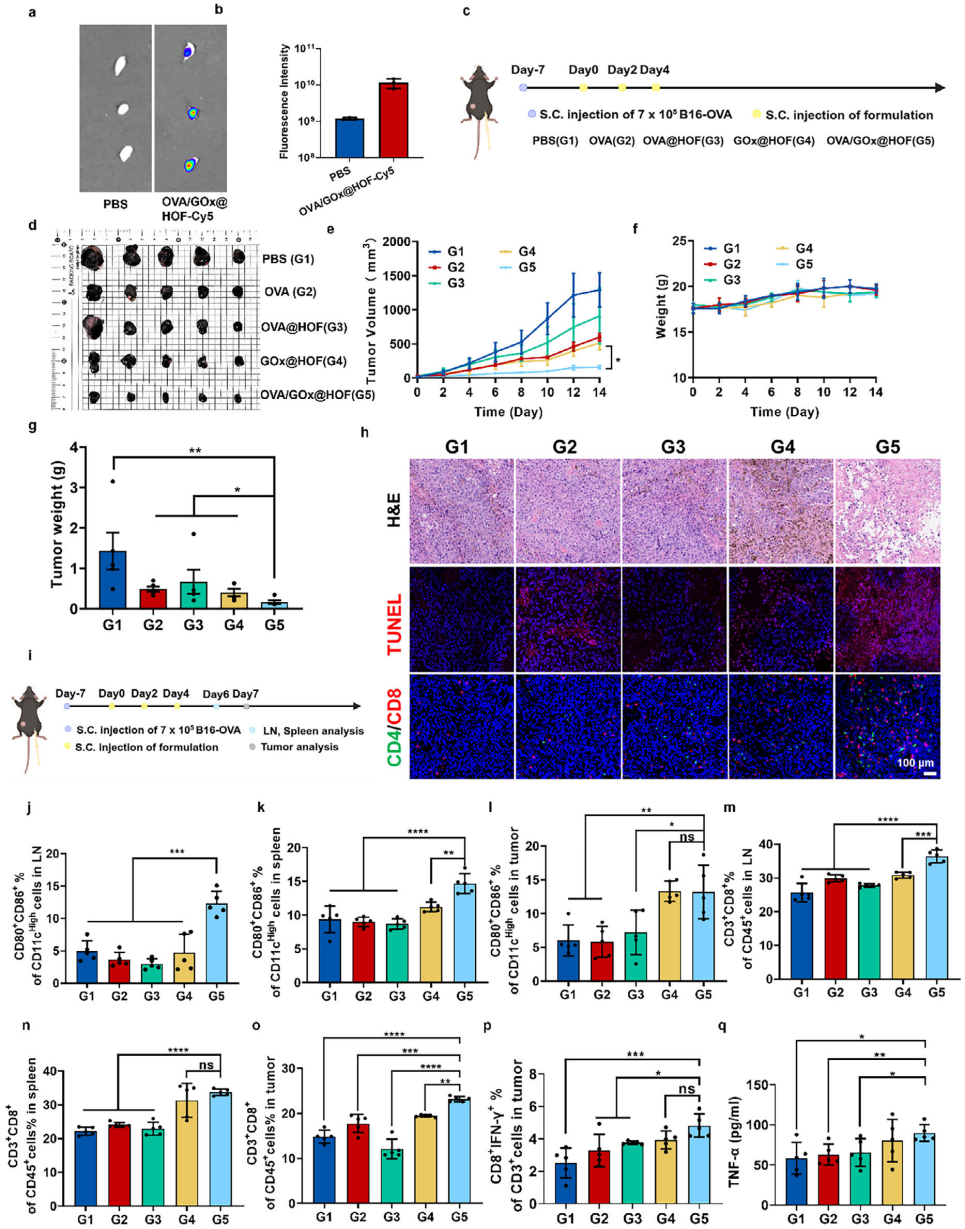
OVA/GOx@HOF promoted cytotoxic T cell response for tumor inhibition. (a) Fluorescence image shows the distribution of Cy5‐labeled OVA/GOx@HOF in the inguinal dLNs 24 h after subcutaneous administration (n = 3). (b) Semiquantification of fluorescence signal in the dLNs ex vivo (n = 3). (c) Flow chart of the in vivo study in a B16‐OVA subcutaneous tumor model. (d) Photograph of resected tumors at the endpoint. (e) Tumor volume curves since first administration (n = 5 mice). (f) Tumor weight of mice from each group (n = 5 mice). (g) Body weight of mice with different treatments (n = 5 mice). (h) Representative histological sections showing H&E staining and immunofluorescence labeling for TUNEL (red) and CD4 (red)/CD8 (green) in tumor tissues following various treatments. (i) Scheme diagram of the immunological analysis in vivo. (j–l) Flow cytometric analysis of CD80^+^CD86^+^ in LN (j), spleen (k), tumor (l) with various treatments (n = 5 mice). (m–o) Flow cytometric analysis of CD3^+^CD8^+^ T cells in LN (m), spleen (n), tumor (o) with various treatments (n = 5 mice). (p) Flow cytometric analysis of CD8^+^IFN‐γ^+^ T cells in tumors with various treatments (n = 5 mice). (q) Cytokine secretion levels of TNF‐α (n = 5 mice). Data are presented in the form of means ± SD. *P* values were determined by ANOVA, ^*^ *p* < 0.05, ^**^ *p* < 0.01, ^***^ *p* < 0.001, and ^****^ *p* < 0.0001.

We subcutaneously inoculated B16‐OVA melanoma cells into the ventral side of C57BL/6 mice and randomly divided them into 5 groups, including PBS, OVA, OVA@HOF, GOx@HOF, and OVA/GOx@HOF to study the in vivo anti‐tumor efficacy of different treatments. The treatment plan is shown in Figure 5c. As shown in Figure 5d–g, compared with the rapid tumor progression in the PBS group, the OVA and GOx@HOF groups showed mild anti‐tumor effects, and OVA/GOx@HOF showed the strongest tumor suppression effect, highlighting the substantial therapeutic effect. Furthermore, the favorable safety profile of the nanobiohybrid vaccines was supported by the absence of significant body weight changes in all mice throughout the treatment period. Hematoxylin and eosin staining (H&E) and terminal deoxynucleotidyl transferase‐mediated dUTP biotin nick end labeling (TUNEL) tumor staining were used to see the tumor morphological changes and apoptosis. As depicted in Figure 5h, the OVA/GOx@HOF group exhibited clear signs of apoptosis, notably nuclear condensation and loss, coupled with widened intercellular spaces between tumor cells. The TUNEL staining image further confirmed extensive apoptosis in tumors. The biocompatibility of OVA/GOx@HOF was performed in the hearts, livers, spleens, lungs, and kidneys (Figure S10). To evaluate the therapeutic efficacy of OVA/GOx@HOF in a more clinically relevant setting, we initiated treatment when B16‐OVA tumors reached ∼100 mm^3^. Mice were subcutaneously injected with PBS, OVA or OVA/GOx@HOF on days 0, 2, and 4. As shown in Figure S24, OVA/GOx@HOF significantly suppressed tumor growth compared to all control groups, with markedly reduced final tumor weights. No significant body weight loss was observed in any group. Although the antitumor effect was slightly attenuated relative to the early‐treatment model, OVA/GOx@HOF antitumor effect remained significant. These results confirm that OVA/GOx@HOF retains potent activity against established, palpable tumors, supporting its translational potential.

To directly assess whether OVA/GOx@HOF modulates DC metabolism and redox homeostasis in vivo, we isolated CD11c^High^ dendritic cells from draining lymph nodes of treated mice. As shown in Figure S19a‐c, DCs from OVA/GOx@HOFtreated mice exhibited significantly lower intracellular G6P levels, a markedly reduced GSH/GSSG ratio, and elevated ROS levels compared to DCs from PBS‐ or OVA‐treated controls. These data provide direct evidence that the nanovaccine specifically targets DCs in vivo and induces the expected metabolic perturbations. Notably, while we attempted to assess LC3‐II conversion in the same sorted DC populations by Western blot, the limited cell yield from lymph nodes precluded reliable detection of the low‐abundance LC3‐II protein. Nevertheless, our robust in vitro findings (Figure 3g,h) and the in vivo metabolic data collectively support that OVA/GOx@HOF induces autophagy–dependent cross‐presentation in DCs.

Then, we analyzed the phenotypes of immune cells 48 h after the last vaccination (Figure 5i). OVA/GOx@HOF significantly promoted the maturation of dendritic cells in lymph nodes. Compared to OVA, OVA/GOx@HOF the percentage of CD11c^High^ DC cell maturation (CD80^+^CD86^+^) induced in lymph nodes, spleen, and tumors increased by 2.4, 1.6, and 2.2 times, indicating OVA/GOx@HOF significantly promoted the maturity of DC (Figure 5j–l; Figures S11 and S27‐S29). Once DCs are activated, the secreted pro‐inflammatory cytokines can further trigger cytotoxic T lymphocytes (CTLs). As shown in Figure 5q and Figure S14, TNF‐α, IL‐6, and IL‐1β cytokines in peripheral blood significantly increased, leading to a significant increase in the frequency of CD8^+^T cells in lymph nodes, spleen, and tumors (Figure 5m–o and Figure S12 and Figures S30–S32). Immunofluorescence staining further indicated that compared with other groups, OVA/GOX@HOF treatment led to the highest infiltration of CD8 T cells in the tumors (Figure 5h), indicating the activation of T cell immunity. In addition, the most potent CTLs response was observed in the OVA/GOX@HOF group (Figure 5p and Figure S13), as evidenced by the highest frequency of CD8^+^IFN‐γ^+^ T cells, confirming successful T cell activation and tumor infiltration.

Interestingly, while OVA/GOx@HOF treatment significantly increased CD4^+^ T cell infiltration in tumors (Figure S21a), the proportion of CD4^+^ T cells in draining lymph nodes was decreased compared to the control group (Figure S21b). This observation is consistent with an effective vaccine‐induced immune response, wherein activated CD4^+^ T cells undergo proliferation and subsequently egress from lymph nodes to peripheral tissues, including the tumor microenvironment. The concurrent increase of CD4^+^ T cells in tumors and their relative decrease in lymph nodes support a process of effector T cell redistribution rather than immunosuppression. Together, these findings demonstrate that OVA/GOx@HOF promotes a coordinated adaptive immune response involving both CD4^+^ and CD8^+^ T cell compartments.

Notably, tumor‐associated macrophages (TAMs) are key immune components present in the tumor microenvironment; However, they predominantly exhibit an M2‐like phenotype in tumors, leading to immunosuppression that promotes tumor progression [45, 46, 47]. We next evaluated whether OVA/GOx@HOF treatment influenced the polarization state of intratumoral macrophages The results showed that, compared with the control group, the OVA/GOx@HOF treatment group exhibited a significant increase in the ratio of M1‐like (F4/80^+^CD86^+^) to M2‐like (F4/80^+^CD206^+^) macrophages, along with enhanced release of the related cytokine TNF‐α (Figures S20 and S33). These findings indicate that the potent adaptive immunity triggered by the vaccine indirectly promotes a more pro‐inflammatory and anti‐tumor tumor microenvironment (TME).

The anti‐metastatic potential of OVA/GOx@HOF was assessed using a lung metastasis model established via intravenous injection of B16‐OVA cells (Figure 6a). As shown in Figure 6b–d, tumor metastasis occurred in the lungs of mice in the control group, mainly manifested as black nodules of different sizes on the lung lobes, lung enlargement, and lung lobe roughness. In the groups of OVA, OVA@HOF, and GOx@HOF, the number of black nodules on the lung lobes of mice gradually decreased, and compared to the control group, the lung lobes were flatter and smoother sutra OVA/GOx@HOF. The mice treated with nanovaccines had the lowest number of metastatic nodules in their lungs and a near‐normal lung morphology. The OVA/GOx@HOF induced inhibition of metastasis was further confirmed by H&E staining of lung tissues. These results evidenced that OVA/GOx@HOF nanobiohybrid vaccines have excellent anti‐metastasis activity.

**FIGURE 6:**
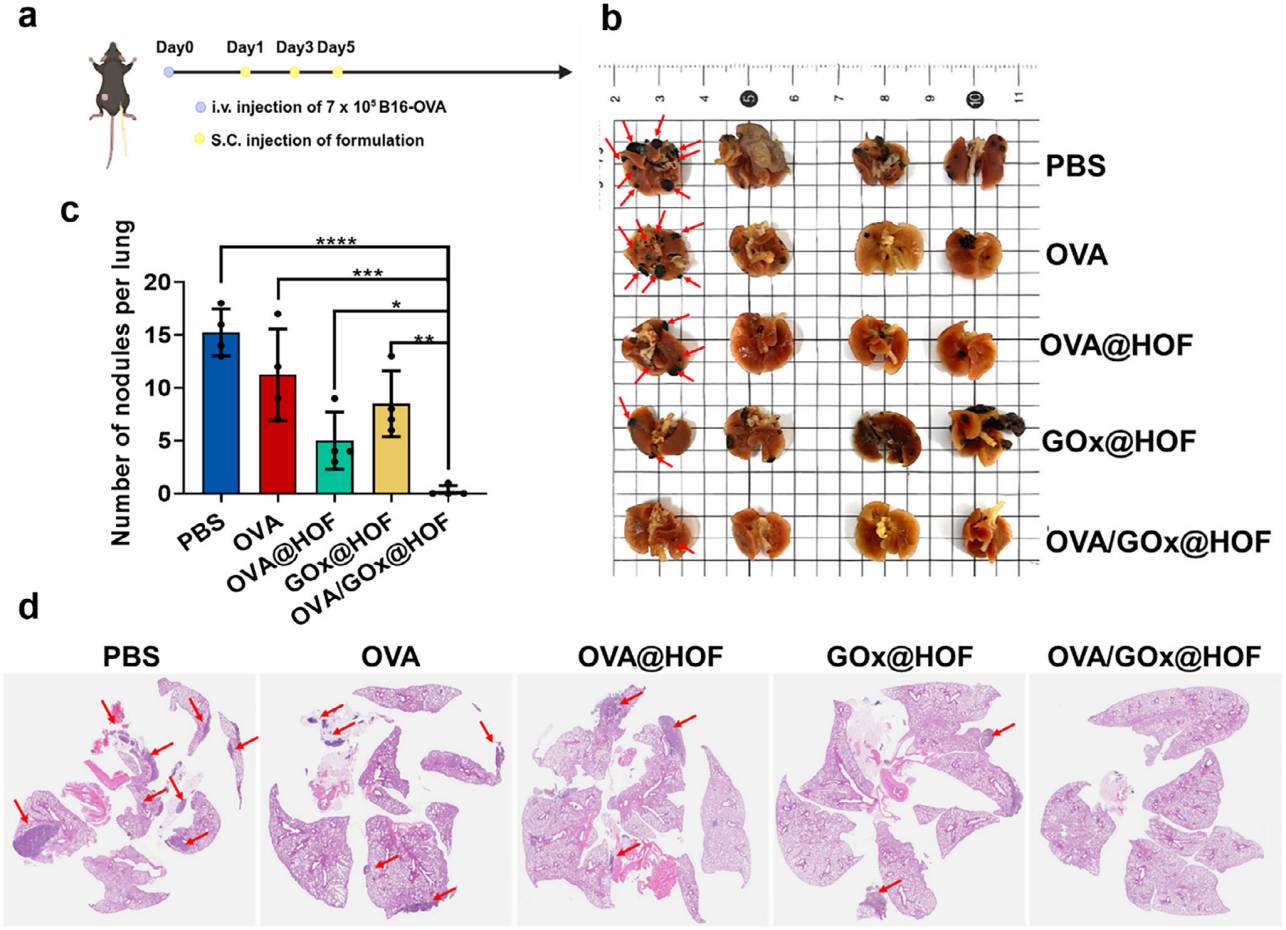
Evaluation of the efficacy of OVA/GOX@HOF in inhibiting lung metastasis (a) Scheme diagram of the timeline for B16‐OVA lung metastasis model establishment and cancer immunotherapy. (b) Photograph of lung specimens collected on day 30 from mice following different treatments. (n = 4). (c) Average number of lung metastatic foci of the B16‐OVA tumor. (d) Representative H&E staining of lung sections from mice in different groups. Data are presented in the form of means ± SD. *P* values were analyzed by one‐way ANOVA, ^*^ *p* < 0.05, ^**^ *p* < 0.01, ^***^ *p* < 0.001, and ^****^ *p* < 0.0001.

To elucidate the immune mechanisms underlying the antimetastatic activity of OVA/GOx@HOF, we analyzed immune cell populations in the spleens of mice from the B16‐OVA lung metastasis model on posttumor inoculation. As shown in Figure S25, OVA/GOx@HOF treatment significantly increased the frequency of mature DCs (CD11c^High^CD80^+^CD86^+^) in the spleen compared to the PBS, OVA groups. Notably, the percentage of CD8^+^ T cells was markedly elevated in the spleen of OVA/GOx@HOFtreated mice. The results show that the antimetastatic effect is mechanistically linked to the vaccine's ability to induce systemic and local T cell immunity.

### 2.5 Nanobiohybrid‐Based Personalized Cancer Vaccine Inhibited Tumor Progression and Exhibited Synergy With Immune Checkpoint Blockade (ICB)

Next, we attempted to validate the applicability of the nanobiohybrid platform as a personalized cancer vaccine. Obsl1 and Tyrp1 peptides were selected as melanoma antigens and were encapsulated in the GOx@HOF (denoted OT/GOx@HOF). A lung metastatic tumor model was established by intravenous injection of B16 tumor cells expressing firefly luciferase (B16‐Luc) into C57BL/6 mice (Figure 7a). The metastasis of B16‐Luc tumor cells was tracked by bioluminescence imaging on days 2, 10, and 20 post‐tumor inoculation (Figure 7b,d). On day 10, weak metastatic signals could be observed in control and Obsl1/Tyrp1 groups but not in OT/GOx@HOF group. On day 20, significant metastasis occurred in the control group. Compared to mice treated with Obsl1/Tyrp1, the treatment of OT/GOx@HOF significantly delayed tumor metastasis and prolonged the survival period of tumor‐bearing mice (Figure 7c). These findings further emphasize the potential of nanobiohybrid personalized vaccine in effectively inhibiting tumor metastasis.

**FIGURE 7:**
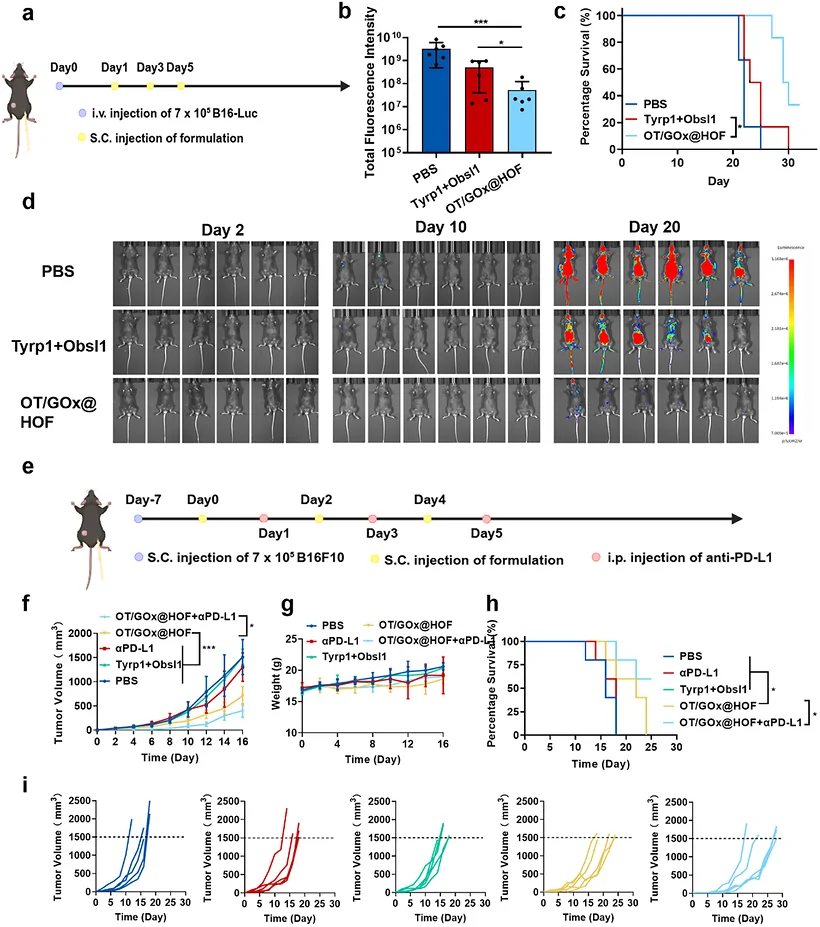
A nanobiohybrid personalized cancer vaccine suppressed tumor progression. (a) Scheme diagram of the timeline for the B16‐Luc lung metastasis model establishment and cancer immunotherapy. (b) Quantitative analysis of bioluminescence images. (c) Survival rate of B16‐Luc lung metastasis model mice with different treatments (n = 6). (d) In vivo bioluminescence images of the B16‐Luc lung metastasis model. (e) Scheme diagram of the experimental timeline for establishing the B16F10 subcutaneous tumor model and administering the immunotherapy protocol. (f) Tumor volume curves since first administration (n = 5). (g) Body weight of mice with different treatments (n = 5). (h) Survival rate of B16F10 tumor‐bearing mice post‐treatments (n = 5). (i) Individual mouse tumor growth profile. Data are presented in the form of means ± SEM. *P* values were analyzed by Student's t‐test and one‐way ANOVA, ^*^ *p* < 0.05, ^**^ *p* < 0.01, and ^***^ *p* < 0.001.

Finally, we explored whether OT/GOx@HOF could synergize with ICB, i.e., αPD‐L1, to further improve the therapeutic effect. A subcutaneous B16F10 tumor model was established by inoculating B16F10 cells into C57BL6 mice (Figure 7e). Upon reaching a tumor volume of approximately 50 mm^3^, the mice were randomized into five experimental groups (PBS, αPD‐L1, Obsl1+Tyrp1, OT/GOx@HOF, and OT/GOx@HOF+αPD‐L1). As shown in Figure 7f,i, OT/GOx@HOF+αPD‐L1 group showed excellent anti‐tumor effects, which significantly outperformed OT/GOx@HOF and αPD‐L1 groups. Moreover, compared to other groups, in the OT/GOX@HOF+αPD‐L1 group on day 25, 60% of the mice were still alive, which effectively prolonged the survival of mice (Figure 7h), indicating that the combination therapy of OT/GOX@HOF nanobiohybrid vaccine and αPD‐L1 effectively inhibits tumor growth. To investigate the immunological basis for the enhanced antitumor activity observed with the combination of OT/GOx@HOF and αPD‐L1, we analyzed the immune cell profiles in draining lymph nodes (dLNs) and tumors from mice treated with PBS, αPD‐L1, OT/GOx@HOF, or the combination. As shown in Figure S26, the combination therapy significantly increased the frequency of mature DCs (CD11c^High^CD80^+^CD86^+^) in dLNs compared to either monotherapy. More importantly, in the tumor microenvironment, flow cytometric analysis revealed that the combination treatment led to a significantly higher infiltration of CD8^+^ T cells compared to all control groups (Figure S26a). These findings demonstrate that OT/GOx@HOF synergizes with αPD‐L1 by promoting DC maturation of lymph nodes, which in turn drives the accumulation of CD8^+^ T cells, overcoming the immunosuppressive tumor microenvironment.

## 3 Conclusion

Dendritic cells (DCs) are the central orchestrators of both innate and adaptive antitumor immunity. As the most potent professional antigen‐presenting cells, DCs possess the unique capacity to capture tumor antigens, migrate to draining lymph nodes, and cross‐present antigens on MHCI molecules to prime naïve CD8^+^ cytotoxic T lymphocytes (CTLs) [35, 48]. This DC‐CTL axis is fundamental for generating durable, systemic antitumor immunity. In contrast, while tumor‐associated macrophages (TAMs) are abundant in the tumor microenvironment (TME) and represent attractive targets for immunomodulation [49, 50, 51], their predominant function in established tumors is often immunosuppressive (M2‐like phenotype), and they lack the specialized machinery for efficient antigen cross‐presentation and T cell priming. Moreover, the subcutaneous administration route used in our study naturally favors DC engagement, as the dermis and hypodermis harbor a dense network of resident and migratory DCs that are specialized for antigen capture and lymph node homing [27, 43, 44]. Our in vivo uptake data (Figure S18) confirmed that OVA/GOx@HOF was predominantly taken up by CD11c^High^ DCs rather than F4/80^+^ macrophages or tumor cells. Thus, while macrophage‐targeted vaccines have shown promise [52, 53], a DC‐centric approach remains the most direct strategy for initiating de novo adaptive antitumor responses.

Several recent studies have explored metabolic reprogramming of DCs to enhance cancer vaccination. For instance, glycolysis inhibition or pentose phosphate pathway (PPP) modulation has been shown to improve DC maturation and T cell priming [54, 55]. However, these approaches typically target a single metabolic node and do not coordinately engage both the autophagic cross‐presentation machinery and innate proinflammatory pathways. In contrast, our OVA/GOx@HOF platform uniquely integrates two complementary mechanisms: (i) GOx‐catalyzed glucose depletion and oxidative stress induce autophagy‐dependent MHC‐I cross‐presentation, which directly amplifies CD8^+^ T cell responses; (ii) simultaneously, the generation of endogenous hydrogen sulfide (H_2_S) potentiates NFκBdriven NLRP3 inflammasome activation, leading to robust DC maturation and IL1β/IL18 secretion. This dual‐action strategy not only augments adaptive immunity but also reinforces the innate inflammatory milieu, a feature not reported in previously described DC metabolism‐targeting vaccines.

Nevertheless, our platform has certain limitations. The reliance on GOx enzymatic activity requires a hypoxic environment for efficient azobond cleavage and payload release, which may vary across different tumor types. Moreover, while we observed secondary repolarization of TAMs toward an M1‐like phenotype (Figure S20), the direct impact of OVA/GOx@HOF on other myeloid cells (e.g., monocytes, MDSCs) remains to be fully characterized. Future optimization could involve engineering HOF scaffolds with tunable hypoxia sensitivity or incorporating additional targeting ligands to further enhance DC specificity. Collectively, this nanobiohybrid strategy highlights the potential of metabolic‐immune crosstalk modulation and provides a versatile platform for next‐generation cancer vaccines.

In summary, this study reported a novel glucose metabolism‐modulatory nanobiohybrid vaccine platform as an effective multi‐faceted immunostimulant for tumor immunotherapy. The catalytic activity of GOx induced intracellular glucose deprivation, which triggers autophagic response and promotes antigen cross‐presentation—a key step for effective CD8^+^ T cell priming. At the same time, the platform triggered intracellular proinflammatory cascades, including the disruption of redox homeostasis and upregulation of sulfur metabolism, resulting in a significant increase in endogenous hydrogen sulfide that enhances the assembly and activation of the NLRP3 inflammasome and the subsequent secretion of the proinflammatory cytokine IL‐1β. The therapeutic effect of this mechanism was demonstrated in vivo, where OVA/GOx@HOF vaccination can induce effective systemic anti‐tumor immunity and effectively inhibit the growth and metastatic progression of established melanoma. Moreover, the compatibility of this platform with neoantigens and synergy with immune checkpoint blockade therapy emphasizes its potential to be integrated into combination treatment regimens for personalized vaccine development and therapy. Thus, this study highlights the promising potential of OVA/GOx@HOF nanobiohybrid materials in cancer vaccines, offering a new avenue in the realm of immunotherapy.

## 4 Experimental Section

### 4.1 Materials

TAM, AZB, and GOx were purchased from Aladdin. Ovalbumin was purchased from InvivoGen. Sulfo‐Cyanine5 (Cy5)‐NHS was purchased from MedChemExpress. Mouse Enzyme‐linked Immunosorbent Assay (ELISA) kits, IL‐6, TNF‐α, IL‐1β, and IFN‐γ were included, which were purchased from MultiSciences Biological Technology Co. Ltd. NLRP3, ASC, and LC3 antibodies were provided by Abcam Biological Technology Co. Ltd (Shanghai, China). Caspase‐1 and NF‐κB antibodies were provided by Cell Signaling Technology. Flow cytometry antibodies and αPD‐L1 were obtained from Biolegend. WSP‐1 and DL‐PAG were provided by MRBIOTECH Co. Ltd.

### 4.2 Instruments

The morphology and size of GOx@HOF, OVA/GOx@HOF, and nanoparticles after degradation were studied using a transmission electron microscope (HT7800 TEM). A microplate reader (BioTek Synergy H1, USA) was used to detect cell viability. The Western blot signals were visualized using Tanon Imaging. Flow cytometry data were obtained via BD LSRFortessa. In vivo imaging of mice was performed by Aniview Kirin in Vivo Image System (Guangzhou Biolight Biotechnology Co., Ltd., Guangzhou, China).

### 4.3 Cells and Animals

All cell lines (B16F10, DC2.4, B16‐OVA, B16‐Luc) were acquired from Hunan Fenghui Biotechnology Co. Ltd. and cultured at 37°C with 5% CO_2_ in RPMI 1640 base medium (Genomcell Bio) containing 10% FBS (Excell) and 1% penicillin/streptomycin (Genomcell Bio). For B16‐Luc cells, the medium was additionally supplemented with 2 µg/mL Puromycin. Female C57BL/6 mice (6‐8 weeks) were obtained from Shanghai BK/KY Biotechnology Co. Ltd. All animal experiments were performed in compliance with the Fudan University Guidelines for Care and Use of Laboratory Animals and were authorized by the Institutional Animal Care and Use Committee of Fudan University (approval no. 202106046S).

### 4.4 Preparation of GOx@HOF and OVA@HOF

The synthesis of HOF was conducted as previously described. For the preparation of GOx@HOF and OVA@HOF, TAM was initially mixed with 0.08 mg of GOx or 0.8 mg of OVA. After 20 min of stirring at room temperature, AZB was introduced (TAM:AZB = 1:4) and the total volume was brought to 880 µL with water. The reaction proceeded for an additional 2 h in the dark before the yellow suspension was centrifuged to obtain the crude product (GOx@HOF or OVA@HOF). The product was then purified through three cycles of water washing and vacuum‐dried overnight at room temperature, yielding a yellow powder.

### 4.5 Preparation of OVA/GOx@HOF and OT/GOx@HOF

OVA/GOX@HOF and OT/GOX@HOF were synthesized following the same procedure as described above, with the only modification being the protein components added to TAM: 0.08 mg GOx along with 0.8 mg of OVA (for OVA/GOX@HOF) or a mixture of 0.2 mg Tyrp1 and Obsl1 (for OT/GOX@HOF). The product, obtained as a yellow solid, was thoroughly washed with deionized water (three times) to ensure the complete removal of any unincorporated starting materials. The final product, obtained as a yellow powder, was collected after drying under vacuum at room temperature overnight.

### 4.6 Preparation of OVA/GOx@HOF‐Cy5

Cy5‐labeling of OVA/GOx@HOF was performed by incubating 5 mg of the composite with 125 µL of 1 mM Cy5 (in DMSO) in 2 mL of 0.1 m NaHCO_3_ overnight in the dark. The resulting green suspension was centrifuged to collect the OVA/GOx@HOF‐Cy5 conjugate, which was then washed with a 1:3 ethanol‐water solution.

### 4.7 Protein Level Assay

Protein encapsulation in the various composites (GOx@HOF, OVA@HOF, OVA/GOx@HOF, OT/GOx@HOF) was assessed via BCA method and HPLC. The following equations were applied to determine the loading capacity (LC) and loading efficiency (LE):

(1) $$\mathrm{LE}\;\left(\%\right)=\frac{w_{1}}{w_{2}}\;\times \;100\%$$

(2) $$\mathrm{LC}\;\left(\%\right)=\frac{w_{1}}{w_{3}}\;\times \;100\%$$

Herein, W1 is defined as the mass of GOx and antigen successfully incorporated into the nanoparticles, W2 refers to the initial mass of GOx and antigen added, and W3 indicates the final nanoparticle mass.

### 4.8 Protein Release of OVA/GOx@HOF‐FITC

The hypoxia‐responsive cleavage of azobenzene moieties was evaluated using OVA/GOx@HOF‐FITC. This composite was dissolved in DI water (2 mg/mL) and incubated for 24 h in the presence or absence of Na_2_S_2_O_4_ (20 mm). Morphological changes induced by the treatment were examined by transmission electron microscopy (TEM). Furthermore, the release profile of OVA‐FITC was monitored by measuring the fluorescence intensity (λex = 488 nm, λem = 525 nm) with a BioTek Synergy H1 microplate reader.

### 4.9 Catalytic Reactivity

To assess catalytic performance, OVA/GOx@HOF and free GOx at equivalent concentrations were separately reacted with glucose solution (1 mg mL^−1^) for 8 h. The pH profile was recorded throughout the reaction period, while solution aliquots collected at various time points were subjected to glucose and H_2_O_2_ quantification using specific commercial kits according to established protocols.

### 4.10 Cytotoxicity Assay

The cytotoxicities of GOx, HOF, GOx@HOF, and OVA/GOx@HOF were assessed via MTT assay. Briefly, DC2.4 cells were plated in 96‐well plates (1 × 10^4^ cells per well) and incubated overnight. Serial dilutions of each material were prepared in low‐glucose RPMI 1640 medium (1 mg mL^−1^ glucose) and applied to the cells. After 24 h of exposure, the medium was exchanged for a fresh low‐glucose medium containing MTT (5 mg mL^−1^). Followed by 4 h of further incubation, the formazan crystals formed were dissolved by the addition of 200 µL DMSO per well. Finally, the absorbance at 570 nm was recorded using a microplate reader to determine cell viability.

### 4.11 DC2.4 Maturation and Cross‐Presentation

To perform DC activation and cross‐presentation assays, immature DC2.4 cells were plated in 6‐well non‐treated plates (Corning) at 3×10^5^ cells/well. To assess DC activation, cells received 24 h treatments with PBS, HOF, GOx@HOF, or OVA/GOx@HOF nanovaccine. Parallel samples were treated with PBS, OVA, OVA@HOF, or OVA/GOx@HOF under identical conditions (37°C, 24 h) for cross‐presentation evaluation. Post‐incubation, all cells were harvested via trypsinization and washing. Before immunostaining, cells were blocked with an Fc block for 15 min at room temperature. DC activation was analyzed using fluorescent antibodies against CD80 and CD86, while cross‐presentation was detected with anti‐SIINFEKL‐H‐2Kb‐APC antibody. All staining procedures were conducted in flow cytometry buffer for 30 min following manufacturer (Thermo Fisher Scientific) protocols, with final analysis performed by flow cytometry. Additionally, cytokine secretion was measured by collecting cell culture supernatants after the 24 h stimulation. The supernatants were centrifuged at 12 000 rpm for 5 min, and the levels of IL‐1β and IL‐6 in the clarified supernatant were determined using commercial ELISA kits (Multi Sciences) in strict accordance with the provided protocol.

### 4.12 Redox Homeostasis

The expression levels of key pentose phosphate pathway (PPP) proteins were assessed to evaluate redox homeostasis disruption. DC2.4 cells were cultured overnight in 6‐well plates at 3 × 10^5^ cells/well before being treated with OVA/GOx@HOF (12.5 µg mL^−1^) in low‐glucose medium for specified time points (0, 3, 6, 12 h). Metabolic parameters including intracellular G6P, GSH, GSSG concentrations, and the NADP^+^/NADPH ratio were measured using corresponding commercial assay kits (Beyotime Biotechnology) following the manufacturer's guidelines. Separately, cells seeded in confocal dishes at 1×10^5^ cells/dish were incubated with nanomaterials (12.5 µg mL^−1^) for 24 h, after which oxidative stress levels were assessed using the DCFH‐DA fluorescent probe.

### 4.13 Western Blot and Immunofluorescence Analysis

Treated DC2.4 cells were harvested for total protein extraction. Western blotting was subsequently employed to analyze the expression of key proteins, including LC3 (an autophagy marker), NLRP3, ASC, caspase‐1, SELENBP1, and NF‐κB. Protein band intensities were quantified with ImageJ software. The formation of autophagosomes and ASC was visualized by immunofluorescence. Briefly, treated cells were fixed with 4% paraformaldehyde and permeabilized with 0.5% Triton X‐100. Cells were then probed with primary antibodies targeting LC3 and ASC, which were subsequently detected using corresponding fluorescently conjugated secondary antibodies.

### 4.14 H_2_S

To monitor the intracellular H_2_S generation, DC 2.4 cells were cultured in a 6‐well plate and treated with various formulations for 24 h: PBS, OVA@HOF, GOx@HOF, OVA/GOx@HOF, and OVA/GOx@HOF+PAG. Following medium removal, the cells were incubated with WSP‐1 for 0.5 h. They were then harvested via trypsinization and rinsing, and subsequently analyzed using both a microplate reader and flow cytometry.

### 4.15 In Vivo Antitumor Activity of OVA/GOx@HOF and OT/GOx@HOF

Therapeutic efficacy was evaluated In a B16‐OVA melanoma model (C57BL/6 mice, 7×10^5^ cells/mouse, left flank). When initial tumors reached ∼50 mm^3^, the mice were randomized into five groups (n = 5): PBS, OVA (0.3 mg/kg), OVA@HOF, GOx@HOF, and OVA/GOx@HOF (2.5 mg/kg). The respective formulations were administered subcutaneously (The vaccine was administered subcutaneously into the inguinal region, contralateral to the tumor site) on days 0, 2, and 4 post‐grouping. Tumor length, width, and body weight were recorded every two days using a Vernier caliper and a scale, respectively, to track tumor growth curves and systemic toxicity. The study endpoint was defined as a tumor volume exceeding 1500 mm^3^, at which point tumors and major organs (heart, liver, spleen, lung, kidneys) were harvested for subsequent histological (H&E) and immunofluorescence (TUNEL, CD4, CD8) analyses.

A melanoma model was established by subcutaneous inoculation of B16F10 cells (7×10^5^/mouse) into the left flank of C57BL/6 mice. When tumor volumes reached approximately 50 mm^3^, the mice were randomized into five treatment groups (n = 5): PBS, αPD‐L1, Tyrp1+Obsl1, OT/GOX@HOF, and OT/GOx@HOF+αPD‐L1. The respective doses were as follows: OT/GOX@HOF at 2.5 mg/kg, antigen at 0.3 mg/kg, and αPD‐L1 at 150 µg/mouse.

The tumor‐bearing mice received subcutaneous injections (The vaccine was administered subcutaneously into the inguinal region, contralateral to the tumor site) of the respective formulations on days 0, 2, and 4, concomitant with intraperitoneal administration of aPD‐L1 antibody on days 1, 3, and 5. Tumor volumes and body weights were monitored every two days as described previously. The experiment was terminated when tumor volume reached 1500 mm^3^ for tissue collection.

### 4.16 Lung Metastasis Study

The lung metastasis model was performed by intravenous injection of 1.5 × 10^5^ B16‐OVA cells in 200 µL PBS into C57BL/6 mice. Mice were divided into three groups randomly, including the PBS, OVA, OVA@HOF, GOx@HOF, and OVA/GOx@HOF (2.5 mg/kg; OVA: 0.3 mg/kg;) (n = 4). (The vaccine was administered subcutaneously into the inguinal region, contralateral to the tumor site). Then, injections were given every 2 days on days 1, 3, and 5 for a total injection number of 3. On the 30th day, the lungs were obtained for H&E staining and photography.

The lung metastasis model was established by intravenous injection of 2.5 × 10^5^ B16F10‐Luc cells in 200 µL PBS into C57BL/6 mice. After two days, mice were randomly divided into three groups based on the bioluminescence intensity in the lungs, including the PBS, Tyrp1+Obsl1, and OT/GOx@HOF (2.5 mg/kg; antigen: 0.3 mg/kg;) (n = 6). Then, injections were given every 2 days on days 1, 3, and 5 for a total injection number of 3. During the treatments, the mice were imaged by the Aniview Kirin in Vivo Image System as described above.

### 4.17 Analysis of Immune Cells

In the B16‐OVA tumor model, the mice were subcutaneous inoculation (The vaccine was administered subcutaneously into the inguinal region, contralateral to the tumor site) three times with OVA/GOx@HOF on days 0, 2, and 4. Immune responses in vivo were evaluated 48 h following the last treatment. Generally, mice were euthanized, and the lymph nodes, spleens, and tumors were immediately excised and processed for flow cytometric analysis. Single‐cell suspensions prepared from the lymph nodes, spleens, and tumors were stained for 30 min to enable the identification of DCs and macrophages. The staining panel included APC‐conjugated CD45, PE‐conjugated CD11c, PE‐Cy7‐conjugated F4/80, PE‐conjugated CD11b, PerCP/Cyanine5.5‐conjugated CD80, FITC‐conjugated CD86, and the Zombie Aqua Fixable Viability Kit.

Besides, tumor tissues were harvested 72 h post‐final treatment, following an analogous procedure. Single‐cell suspensions derived from these tumors were then subjected to a 30 min immunostaining in flow cytometry buffer for effector T cell profiling. The staining panel included: APC‐anti‐CD45, FITC‐anti‐CD3, PE‐anti‐CD4, PerCP/Cyanine5.5‐anti‐CD8, Brilliant Violet 421‐anti‐IFN‐γ, and the Zombie Aqua Fixable Viability Kit.

Moreover, circulating concentrations of the proinflammatory cytokines IL‐1β, IL‐6, and TNF‐α in mouse serum were determined using corresponding commercial ELISA kits, strictly adhering to the manufacturers' standardized protocols.

### 4.18 In Vivo Cellular Uptake Assay

To identify the cellular targets of OVA/GOx@HOF in vivo, OVA/GOx@HOF‐Cy5 was prepared as described above. C57BL/6 mice bearing B16‐OVA tumors (∼100 mm^3^) were subcutaneously injected. In the B16‐OVA tumor model, the mice were subcutaneously inoculated (The vaccine was administered subcutaneously into the inguinal region, contralateral to the tumor site) two times with OVA/GOx@HOF on days 0 and 2. After 24 h, the mice were euthanized, and the draining lymph nodes and tumors were immediately excised and processed for flow cytometric analysis. Single‐cell suspensions prepared from the lymph nodes and tumors were stained for 30 min to enable the identification of DCs and macrophages. The staining panel included FITC‐conjugated CD45, APC‐conjugated CD45, PE‐conjugated CD11c, BV421‐conjugated Ly6G, FITC‐conjugated CD11b, PE‐conjugated F4/80, APC Rat IgG2b, κ Isotype Ctrl, PE Rat IgG2a, κ Isotype Ctrl, FITC Rat IgG2b, κ Isotype Ctrl, Brilliant Violet 421T Rat IgG2a, κ Isotype Ctrl, and the Zombie Aqua Fixable Viability Kit.

### 4.19 BMDC Extraction

Bone marrow‐derived dendritic cells (BM‐DCs) from C57BL6 mice were cultured according to the method of Manfred B. Lutz [56]. Bone marrow cells were obtained from 6–10‐week‐old C57 mice. Mice were euthanized by cervical dislocation, and femurs and tibiae were harvested. Both epiphyses were cut, and the bone marrow was flushed with PBS using a syringe. The cell suspension was filtered through a 300‐µm nylon mesh and centrifuged at 1200 rpm for 5 min. The pelleted cells were washed once with PBS and resuspended in RPMI1640 medium containing 10% FBS and 1% penicillin/streptomycin.

For BMDC generation, bone marrow cells were counted, selectively enumerating DC progenitor cells (large and bright cells under microscopy). The cell density was adjusted to 1–2 × 10^6^ cells/mL in complete medium supplemented with recombinant mouse GM‐CSF (20 ng/mL) and IL‐4 (10 ng/mL). Cells were seeded in 100mm bacterial‐grade Petri dishes (8 mL/dish) and incubated at 37 °C with 5% CO_2_. On day 3, an additional 8 mL of complete medium containing 20 ng/mL GM‐CSF and IL‐4 (10 ng/mL) was added. On days 6 and 8, half of the medium was replaced by centrifugation and resuspension of the collected cells in fresh complete medium with GM‐CSF (20 ng/mL) and IL‐4 (10 ng/mL). On day 10, both suspended and loosely adherent cells were harvested as BMDCs. Purity was verified by flow cytometry using live/dead discrimination and staining for CD45 and CD11c.

### 4.20 Isolation of Dendritic Cells From Draining Lymph Nodes for Metabolic Analysis

To assess whether OVA/GOx@HOF directly modulates DC metabolism in vivo, CD11c^+^ DCs were isolated from dLNs of treated mice. B16‐OVA tumor‐bearing mice were subcutaneously injected with PBS, OVA (0.3 mg/kg), or OVA/GOx@HOF (2.5 mg/kg) on days 0, 2, and 4 (n = 5 mice per group). On day 5, dLNs were collected from each group and pooled to obtain sufficient cell numbers. Single‐cell suspensions were prepared as described above. The isolation process of CD11c^+^ cells is completed by MultiSciences Biological Technology Co. Ltd. The obtained DCs were then subjected to the following assays.

### 4.21 Statistical Analysis

All data are presented as means ± SD or SEM as indicated. Statistical comparisons were performed using Student's t‐test for two groups or oneway/twoway analysis of variance (ANOVA) with appropriate post hoc tests (Tukey or Bonferroni) for multiple comparisons. A *p* value of <0.05 was considered statistically significant. All statistical analyses were conducted using GraphPad Prism. The criterion was expressed as ^*^ *p* < 0.05, ^**^ *p* < 0.01, ^***^ *p* < 0.001, and ^****^ *p* < 0.0001, and *p* > 0.05 represents a non‐significant difference (ns).

## Author Contributions

**Tianze Wu**: methodology, investigation. **Weidong Wang**: methodology, validation, investigation, writing – original draft. **Jianing Li**: methodology, investigation. **Mingli Deng**: conceptualization, supervision, writing – review and editing, funding acquisition, resources. **Yimin Gong**: data curation, investigation, validation. **Yannan Yang**: conceptualization, writing – review and editing, supervision, resources.

## Conflicts of Interest

The authors declare no conflicts of interest.

## Supporting information




**Supporting File**: advs76654‐sup‐0001‐SuppMat.docx.

## Data Availability

The data that support the findings of this study are available from the corresponding author upon reasonable request.
