# Supplementary material for: A Glucose Metabolism‐Modulatory Nanobiohybrid Vaccine Platform Promotes Anti‐Tumor Immunity by Orchestrating Autophagy‐Dependent Cross‐Presentation and H2S‐Enhanced NLRP3 Inflammasome Signaling
Source: Adv Sci (Weinh). 2026 Jul 21;13(57):e76654. doi: 10.1002/advs.76654 (PMC13386600; doi:10.1002/advs.76654)
Supplement: Supplementary file 1 [file ADVS-13-e76654-s001.docx]

Supporting Information

A glucose metabolism-modulatory nanobiohybrid vaccine platform promotes anti-tumor immunity by orchestrating autophagy-dependent cross presentation and H_2_S-enhanced NLRP3 inflammasome signaling

Weidong Wang, Yimin Gong, Jianing Li, Tianze Wu, Mingli Deng*, and Yannan Yang*

W. Wang, Y. Gong, J. Li, M. Deng

Shanghai Key Laboratory of Molecular Catalysis and Innovative Materials, Department of Chemistry, Fudan University, Shanghai, 200433, China
E-mail: mldeng@fudan.edu.cn

T. Wu

Obstetrics and Gynecology Hospital, State Key Laboratory of Genetics and Development of Complex Phenotypes, Children’s Hospital, Fudan University, Shanghai 200011, China.

Y. Yang
South Australian immunoGENomics Cancer Institute, The University of Adelaide, Adelaide, South Australia, 5005, Australia

E-mail: yannan.yang@adelaide.edu.au

| **Table 1. The loading efficiency of GOx and functional protein.** | | | |
| --- | --- | --- | --- |
| **Name** | | **Loading capacity of GOx (%)** | **Loading efficiency of functional proteins (%)** |
| **OVA/GOx@HOF** | | 5.3 | 13.1 |
| **OT/GOx@HOF** | | 5.3 | 13.6 |
| Table 2. Peptide Sequences | | | |
| Name | Sequence (from N to C) | | |
| Obsl1 | REGVELNKYEMRRHGTTHSLVIHD | | |
| Tyrp1 | TAPDNLGYM | | |

**Figure S1.** HPLC chromatograms. a) OVA control, b) GOx, c) OVA/GOx@HOF.

**Figure S2.** TEM images of OVA/GOx@HOF before and after Na_2_S_2_O_4_ treatment (20 mM).

**Figure S3.** OVA-FITC release from OVA/GOx@HOF in PBS (pH=7.4) with or without Na_2_S_2_O_4_ (20 mM).

**Figure S4.** Cytotoxicity studies of DC2.4 cells cultivating with HOF, OVA@HOF, GOx@HOF and OVA/GOx@HOF, respectively, using low concentration of glucose (1 mg mL^−1^) for 24 h.

**Figure S5.** Flow cytometry analysis and the corresponding quantification of CD80, CD86, MHC-Ⅰ (G1:OVA/GOx@HOF, G2:GOx@HOF, G3:HOF/OVA, G4:PBS).

**Figure S6**. Statistical data showing OA staining efficiencies in DC2.4 cells treated with NPs for 24 h.

**Figure S7.** Confocal images exhibiting the endosomal escape of OVA-FITC, OVA@HOF, and OVA/GOx@HOF (10 mg mL^−1^) nanoparticles in DC2.4 cells; the lysosomes were stained with Lyso-Tracker Red probe, shown in red; and the nucleus was stained with Hoechst, shown in blue; nanoparticles was shown in green.

**Figure S8.** Flow cytometric analysis of CD80 in DC2.4 cells after incubating with various formulations for 24 h treatment.

**Figure S9.** IL-6 concentrations in supernatants of DC2.4 cells after incubating with various formulations for 24 h treatment.

**Figure S10.** H_2_S detection. a, b) Flow cytometric analysis of H_2_S level in DC2.4 cells after incubating with various formulations for 24 h. c) Statistical analysis of fluorescence intensity of WSP-1 probe detected by microplate reader.

**Figure S11.** Representative H&E staining images of major organs from PBS,OVA, OVA@HOF, GOx@HOF and OVA/GOx@HOF groups, repectively.

**Figure S12.** Representative flow cytometry dot plots of CD80^+^CD86^+^ cells in LN (a), spleen (b) and tumor (c) (G1:PBS, G2:OVA, G3:OVA@HOF, G4:GOx@HOF, G5:OVA/GOx@HOF).

**Figure S13.** Representative flow cytometry dot plots of CD4^+^,CD8^+^ T cells in LN (a), spleen (b) and tumor (c) (G1:PBS, G2:OVA, G3:OVA@HOF, G4:GOx@HOF, G5:OVA/GOx@HOF).

**Figure S14.** Representative flow cytometry dot plots of CD8^+^IFN-γ^+^ T cells in tumor ( (G1:PBS, G2:OVA, G3:OVA@HOF, G4:GOx@HOF, G5:OVA/GOx@HOF).

**Figure S15.** IL-1β,IL-6 concentrations in supernatants of DC2.4 cells after incubating with various formulations for 24 h.

**Figure S16.** Flow cytometry measurement of the mean fluorescence intensity (MFI) of SIINFEKL-H-2Kb (a), CD80 (b), CD86 (c). Data are presented as means ± SEM (n = 3). P values were determined by one‑way ANOVA; **P* < 0.05 ***P* < 0.01, ****P* < 0.001, *****P* < 0.0001.

**Figure S17.** Validation of OVA/GOx@HOF effects in primary bone marrow‑derived dendritic cells (BMDCs). a) Intracellular G6P concentration. b) GSH/GSSG ratio. c) NADPH/NADP⁺ ratio. d) Confocal microscopy images showing intracellular ROS levels in BMDC cells following 24 h treatment with OVA/GOx@HOF. e,f) Flow cytometric analysis of CD80, CD86 (e) and SIINFEKL–H‑2Kb complex (f) level in BMDC after incubated with different formulations after 24 h treatment (n = 3). g) Representative Western blot of LC3‑I and LC3‑II conversion in BMDCs treated with different formulations. Data are presented as means ± SD. P values were determined by one‑way ANOVA; **P* < 0.05 ***P* < 0.01, ****P* < 0.001, *****P* < 0.0001.

**Figure S18.** Cellular GSH/GSSG ratio at indicated time points after OVA/GOx@HOF treatment.

**Figure S19.** In vivo uptake of OVA/GOx@HOF-Cy5 in the lymph nodes (a)and tumors (b).

**Figure S20.** OVA/GOx@HOF modulates glucose metabolism and intracellular redox homeostasis in dendritic cells within lymph nodes. a) Intracellular G6P concentration after various treatment (n = 3). b) ratio of GSH/GSSG after various treatment (n = 3). c) Confocal microscopy images showing intracellular ROS levels after various treatment. Data are presented as means ± SD of three technical replicates from pooled samples. P values were determined by one‑way ANOVA; **P* < 0.01, ***P* < 0.001

**Figure S21.** In vivo characterization of tumor-associated macrophages (TAM). a, b) Flow cytometric analysis of CD86 and CD206 expression in TAM (n = 5). c) Cytokine secretion levels of TNF-α(n = 5). Data are presented as means ± SD. P values were determined by one‑way ANOVA; **P* < 0.05 ***P* < 0.01, ****P* < 0.001.

**Figure S22.** Flow cytometric analysis of CD3^+^CD4^+^ T cells in tumors and LN with various treatments (n = 5). Data are presented as means ± SD. P values were determined by Student’s t‑test; **P* < 0.05 ***P* < 0.01, ****P* < 0.001.

**Figure S23.** Flow cytometry analysis and the corresponding quantification of CD80^+^ (a), CD86^+^ (b) CD40^+^ (c), MHC-Ⅱ^+^ (d) in DC2.4 treated with various treatments for 24 h (n = 3). Data are presented as means ± SD. P values were determined by one‑way ANOVA; **P* < 0.05 ***P* < 0.01, ****P* < 0.001, *****P* < 0.0001. n.s., not significant.

**Figure S24.** a-e) Flow cytometry analysis and the corresponding quantification of CD80^+^ (a), CD86^+^ (b) CD40^+^ (c), MHC-Ⅱ^+^ (d) and OVA antigen cross-presentation level (e) in DC2.4 treated with various treatments for 24 h (n = 3). f) Representative Western blot images reveal NLRP3, ASC, and Caspase-1 expression in DC2.4 cells following incubation with different formulations. Data are presented as means ± SD. P values were determined by one‑way ANOVA; **P* < 0.05 ***P* < 0.01, ****P* < 0.001, *****P* < 0.0001.

**Figure S25.** a) Schematic illustration of the antitumor strategy in the B16-OVA subcutaneous tumor model. b-e) Tumor growth curve(b), photograph(c), body weight(d) and tumor weight(e) of mice with different treatments (n = 5 mice). Data are presented in the form of means ± SEM. P values were analyzed by one-way ANOVA with a Tukey post hoc test, **P* < 0.05 ***P* < 0.01, ****P* < 0.001.

**Figure S26.** a) Flow cytometric analysis of, CD3^+^CD8^+^ T cells in spleen with various treatments (n = 5 mice). b) Flow cytometric analysis of, CD80^+^CD86^+^ in lymph nodes with various treatments (n = 5 mice). Data are presented as means ± SD. P values were determined by one‑way ANOVA; **P* < 0.05 ***P* < 0.01, ****P* < 0.001.

**Figure S27.** a) Flow cytometric analysis of, CD3^+^CD8^+^ T cells in B16-OVA tumors with various treatments (n = 5 mice). b) Flow cytometric analysis of CD80^+^CD86^+^ in B16-OVA tumors with various treatments (n = 5 mice). c) Flow cytometric analysis of, CD80^+^CD86^+^ in lymph nodes with various treatments (n = 5 mice). Data are presented as means ± SD. P values were determined by one‑way ANOVA; **P* < 0.05 ***P* < 0.01, ****P* < 0.001, *****P* < 0.0001.

**Figure S28.** Flow cytometry gating strategy for the analysis of CD80 and CD86 of DCs in LN in vivo.

**Figure S29.** Flow cytometry gating strategy for the analysis of CD80 and CD86 of DCs in spleen in vivo.

**Figure S30.** Flow cytometry gating strategy for the analysis of CD80 and CD86 of DCs in tumor in vivo.

**Figure S31.** Flow cytometry gating strategy for the analysis of CD3^+^CD4^+^ T cells and CD3^+^CD8^+^T cells in the LN in vivo.

**Figure S32.** Flow cytometry gating strategy for the analysis of CD3^+^CD8^+^T cells in the spleen in vivo.

**Figure S33.** Flow cytometry gating strategy for the analysis of CD3^+^CD4^+^ T cells, CD3^+^CD8^+^T cells and CD8^+^IFN^+^ T in the tumor in vivo.

**Figure S34.** Flow cytometry gating strategy for the analysis of CD86^+^ and CD206^+^of TAM in the tumor in vivo.

**Figure S35.** Flow cytometry gating strategy for the analysis of cell uptake of CD11c^High^ DCs cells in LN in vivo.

**Figure S36.** Flow cytometry gating strategy for the analysis of cell uptake of F4/80^+^microphages cells in LN in vivo.

**Figure S37.** Flow cytometry gating strategy for the analysis of cell uptake of CD45^+^F4/80^+^ microphages cells and CD45^-^ tumor cells in tumor in vivo.
